# Applying convergent parallel mixed methods design to investigate medication adherence among people living with HIV in resource-limited settings: methodological insights from Ghana

**DOI:** 10.3389/fpubh.2026.1776541

**Published:** 2026-05-07

**Authors:** Victor Luckyboy Dzramado, Emmanuel Oduro, Obed U. Lasim

**Affiliations:** 1 Department of Biostatistics, Cape Coast Teaching Hospital, Cape Coast, Ghana; 2 National HIV/AIDS Control Program, Kumasi, Ghana; 3 Department of Health Information Management, University of Cape Coast, Cape Coast, Ghana

**Keywords:** antiretroviral therapy, convergent parallel design, Ghana, HIV, medication adherence, mixed methods research, resource-limited settings

## Abstract

**Background:**

Suboptimal adherence to antiretroviral therapy (ART) remains a critical challenge in achieving viral suppression among people living with HIV in resource-limited settings. This study demonstrates the application of convergent parallel mixed methods design to investigate adherence barriers among patients who experienced at least one missed clinic appointment but successfully re-engaged in care.

**Methods:**

We employed a convergent parallel mixed methods design combining quantitative survey data from 288 participants with seven-day pill count adherence measurements across 36 healthcare facilities in Ghana’s Ashanti Region, with qualitative in-depth interviews from 20 participants (10 patients, 10 healthcare providers). Multilevel regression examined associations between demographic factors, self-efficacy, social support, economic factors, and other theoretical constructs with adherence rates, accounting for facility clustering. Thematic analysis of qualitative data identified adherence barriers and facilitators, with findings integrated through joint display matrices.

**Results:**

Mean adherence rates were 80.29% (SD = 20.55%) for efavirenz-based regimens, 79.00% (SD = 22.73%) for dolutegravir-based regimens, and 78.58% (SD = 23.06%) for zidovudine-based regimens, all falling below the 95% threshold for optimal viral suppression. Multilevel regression revealed significant associations with gender, education, employment, and theoretical constructs, though severe multicollinearity (VIF up to 379.19) limits causal interpretation. Qualitative analysis identified five themes: economic barriers, HIV-related stigma, healthcare facility factors, individual motivation, and social support. Integration revealed convergence for economic barriers and healthcare facility factors.

**Conclusion:**

This methodological demonstration illustrates rigorous integration approaches for convergent parallel designs. Findings suggest multilevel barriers requiring comprehensive interventions, pending confirmation in larger representative samples with linkage to clinical outcomes including viral load suppression.

## 1 Introduction

### 1.1 Global and regional context

Antiretroviral therapy has transformed HIV from a fatal diagnosis into a manageable chronic condition, with life expectancy for people living with HIV approaching that of the general population when viral suppression is achieved (1, 2). However, achieving and maintaining viral suppression requires consistent medication adherence, a threshold that remains elusive for many patients, particularly in resource-limited settings (3, 4). The global burden of HIV continues to challenge public health systems, with UNAIDS reporting persistent gaps in the 95–95-95 targets (5).

Several key concepts require operational definition before proceeding. Medication adherence is defined as the extent to which patients take medications as prescribed by their healthcare providers, typically measured as the percentage of prescribed doses actually consumed over a specified period (6). For antiretroviral therapy, adherence rates of 95% or higher have historically been considered necessary to achieve and maintain viral suppression, corresponding to missing fewer than three doses per month on a 30-day supply (7, 8). Viral load suppression is defined in this study according to the World Health Organization 2023 guidelines as plasma HIV RNA less than 1,000 copies/mL after at least 6 months of antiretroviral therapy, indicating that medication is working effectively and significantly reducing transmission risk (9). The WHO further distinguishes viral load suppression from undetectable viral load, the latter typically defined as less than 50 copies/mL using standard laboratory assays and representing the highest level of treatment success (9). People living with HIV who achieve undetectable viral loads through consistent ART use do not transmit HIV to sexual partners and are at low risk of vertical transmission (1, 9). Convergent parallel mixed methods design refers to a research approach in which quantitative and qualitative data are collected simultaneously and independently, analyzed separately, and then integrated during interpretation to provide a more comprehensive understanding of the research problem than either approach alone could yield (10–14). At adherence levels below 90%, virologic suppression rates fall substantially below 50%, markedly increasing risks of treatment failure, drug resistance, disease progression, and ongoing HIV transmission (3, 4, 15).

Viral load suppression is multifactorial and influenced not only by adherence but also by baseline immune status at ART initiation, antiretroviral regimen potency and resistance profile, individual pharmacogenetics, concurrent infections, and nutritional status (16, 17). Consequently, while high adherence is necessary for viral suppression in most patients, it is not always sufficient, and individual variation exists in the adherence threshold required for virologic success (7, 8, 15).

Sub-Saharan Africa bears 67% of the global HIV burden with 25.6 million people living with HIV, yet adherence rates frequently fall below optimal levels, compromising both individual health outcomes and population-level transmission dynamics (5, 18). Ghana, with an estimated 350,000 people living with HIV and adult prevalence of 1.7%, exemplifies these challenges (19, 20). Despite achieving 85% ART coverage among diagnosed individuals, national surveys indicate that only 62% achieve viral suppression, suggesting substantial adherence gaps (21). The Ashanti Region, Ghana’s most populous region with over 5 million inhabitants, reports HIV prevalence of 2.3% and serves as a critical sentinel site for understanding adherence dynamics in West African contexts (21, 22).

### 1.2 Adherence challenges in resource-limited settings

Adherence determinants in resource-limited settings extend beyond individual factors to encompass complex interactions between socioeconomic conditions, healthcare system characteristics, and sociocultural contexts (23, 24). Economic barriers including transportation costs, opportunity costs from missed work, and food insecurity create structural impediments that individual motivation alone cannot overcome (25–28). Healthcare system factors such as medication stock-outs, inflexible clinic hours, long wait times, and provider attitudes shape adherence patterns through both direct access mechanisms and indirect effects on patient trust and engagement (29–32).

HIV-related stigma operates at multiple levels: internalized stigma affects disclosure decisions and medication-taking visibility; interpersonal stigma from family, community, and workplace creates concrete barriers to adherence; and structural stigma embedded in healthcare practices may deter clinic attendance (33–38, 150–155). These multilevel barriers interact in ways that simple additive models fail to capture, necessitating research approaches that can examine both individual-level associations and contextual mechanisms (39–42).

Food insecurity represents a particularly severe barrier in resource-constrained settings, as antiretroviral medications often cause gastrointestinal side effects that are exacerbated when taken without adequate nutrition (28, 43–47). Transportation costs and distance to healthcare facilities create access barriers that disproportionately affect rural and peri-urban populations (25, 27). The economic burden of HIV care extends beyond direct medical costs to include indirect costs such as lost wages from clinic attendance and caregiver time (48, 49).

Social support systems play critical protective roles in adherence maintenance, with treatment supporters, disclosure to family members, and peer support groups facilitating medication-taking behavior (50–55). Conversely, lack of social support compounds adherence challenges through isolation, lack of practical assistance, and absence of adherence monitoring (56–58).

### 1.3 Study population scope

This study focuses specifically on understanding adherence patterns among patients with documented missed clinic appointments who subsequently re-engaged in care. This population differs fundamentally from both consistently adherent patients and those lost to follow-up (40, 41, 59, 60, 156). Patients who miss appointments but return demonstrate partial adherence challenges and continued healthcare engagement, making them an important subgroup for intervention development (41, 61, 62). However, findings cannot be extrapolated to estimate population-level adherence rates or to characterize all people living with HIV in the region.

### 1.4 Theoretical framework

**Figure 1:**
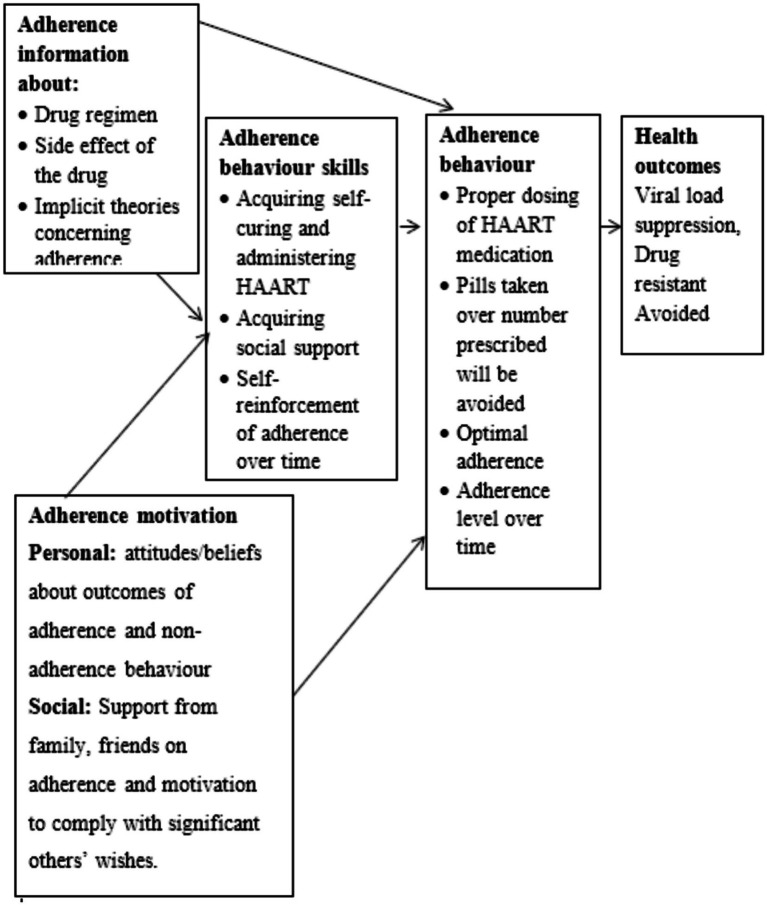
Information motivation behavior model.

**Figure 2:**
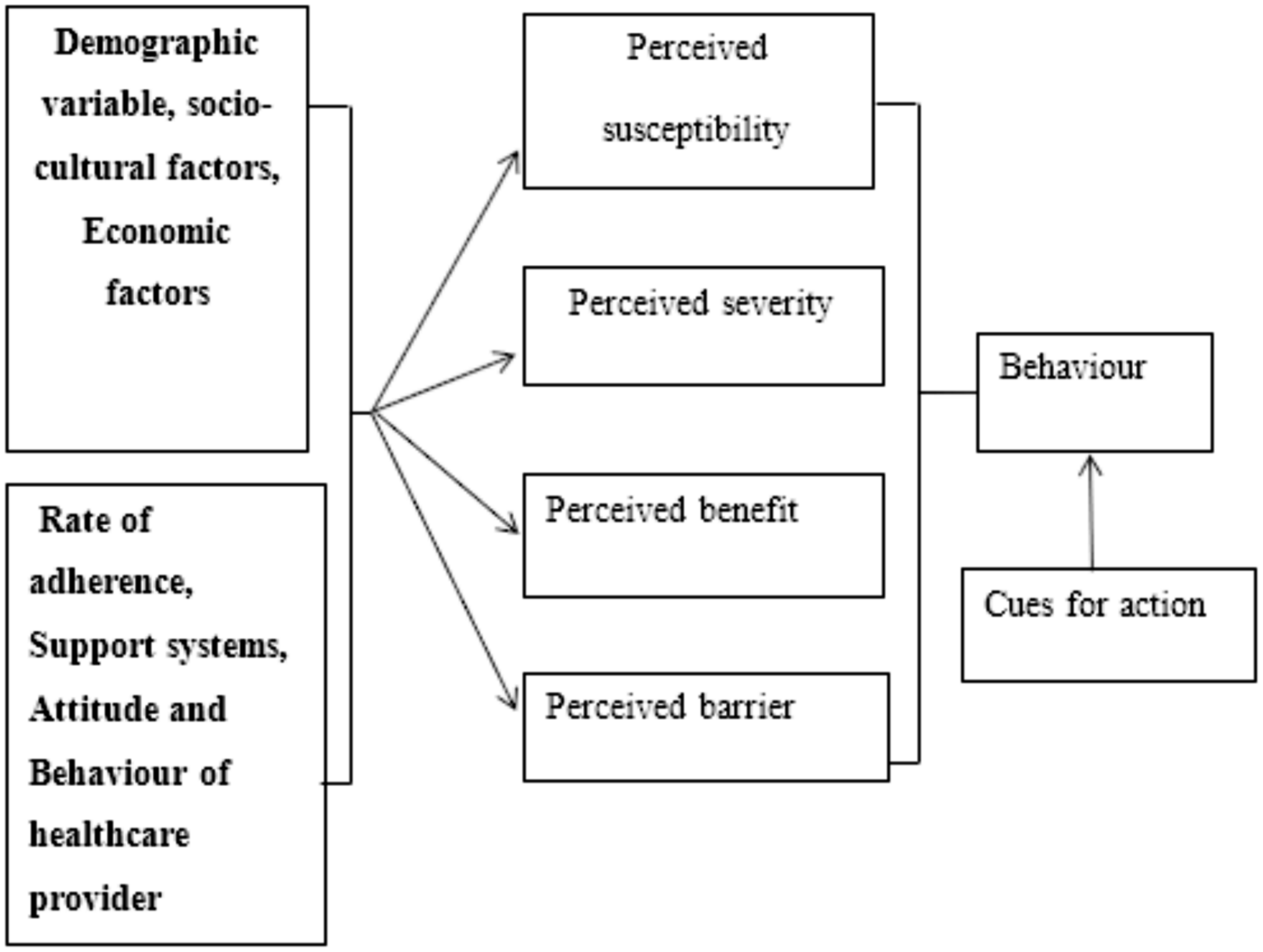
Health belief model.

**Figure 3:**
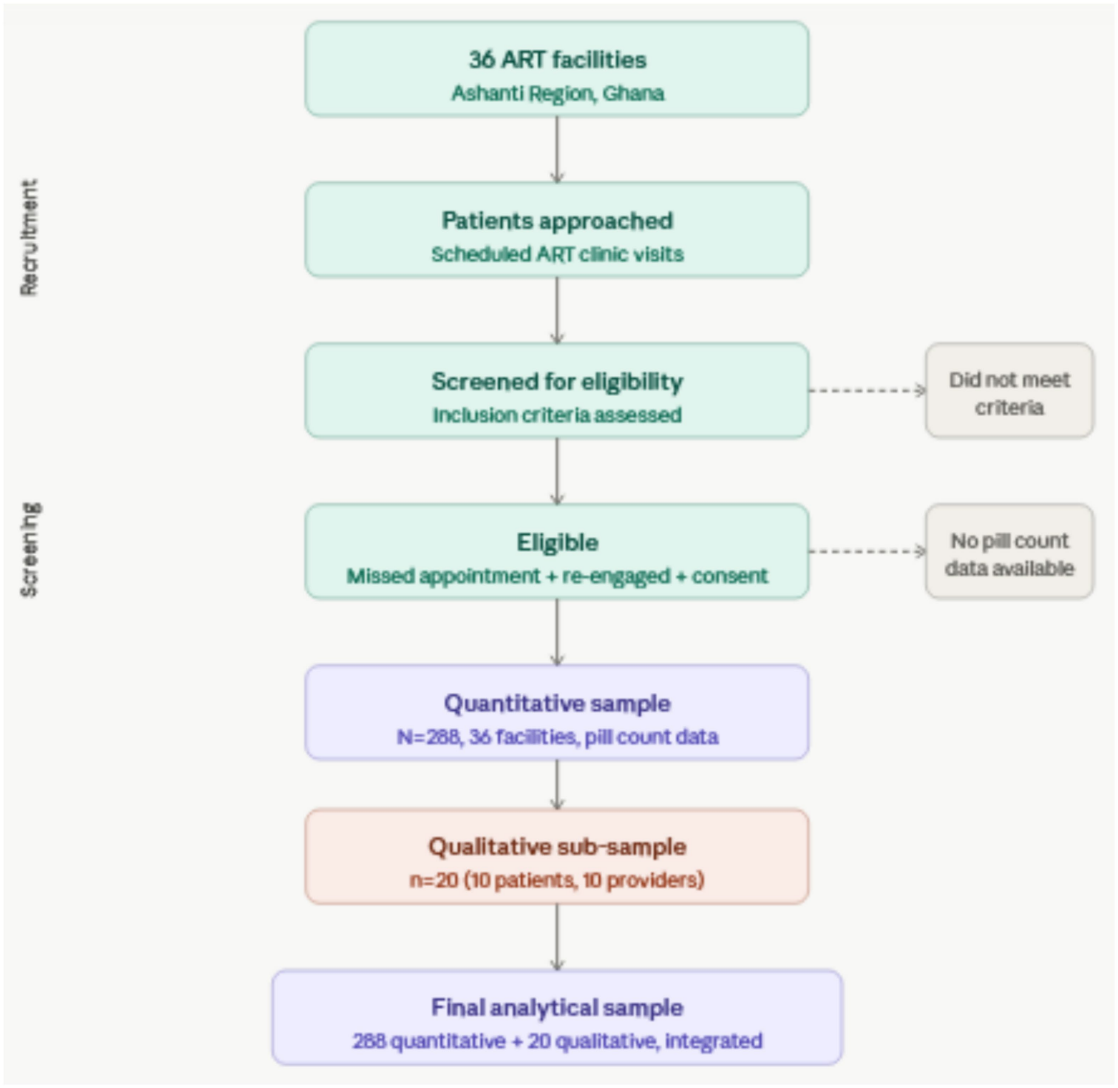
Participant flow diagram.

We integrated two complementary theoretical models to guide investigation. The Information-Motivation-Behavioral Skills (IMB) model posits that adherence behavior results from adequate information about treatment, sufficient motivation to adhere, and behavioral skills necessary for consistent medication-taking (63, 64). This model has demonstrated utility in HIV adherence research across diverse global contexts, including sub-Saharan Africa (65, 66). The Health Belief Model (HBM) contributes understanding of how perceived susceptibility to illness, perceived severity of consequences, perceived benefits of treatment, perceived barriers, cues to action, and self-efficacy influence health behaviors (67–70) (see Figures 1–3).

These frameworks complemented each other: IMB provided structure for examining individual-level cognitive and skill factors, while HBM illuminated how perceptions and beliefs mediate behavioral responses to structural barriers (69–72). Both models emphasize that adherence results from interactions between individual characteristics and environmental contexts, supporting our mixed methods approach (24, 73).

### 1.5 Mixed methods rationale

Quantitative research effectively identifies associations and quantifies effect magnitudes but may miss contextual mechanisms underlying observed patterns (74, 75). Qualitative research reveals nuanced experiences and contextual factors but cannot assess prevalence or statistical significance (76, 77). Convergent parallel mixed methods design collects both data types simultaneously and independently, then integrates findings to leverage complementary strengths while offsetting individual limitations (10–14). This approach enables examination of whether quantitative patterns converge with qualitative themes (convergence), whether qualitative data explains quantitative findings (expansion), and how to reconcile apparent contradictions (divergence) (78–83).

Previous HIV adherence research in sub-Saharan Africa has predominantly employed either quantitative surveys or qualitative interviews separately (59, 84–88). While some studies have used sequential designs where qualitative findings inform quantitative instruments, few have employed truly parallel designs with systematic integration frameworks (85, 89–91). This study contributes methodological demonstration of convergent parallel design implementation, including explicit integration strategies and transparent acknowledgment of limitations when quantitative and qualitative components yield different levels of generalizability (12, 82, 83, 92).

### 1.6 Study objectives

This study aimed to:

- Examine associations between demographic factors and adherence rates, with particular attention to self-efficacy, social support, and economic factors as theoretical constructs, among patients with documented missed appointments who re-engaged in care.
- Investigate patient and provider perspectives on adherence barriers and facilitators through qualitative inquiry.
- Demonstrate rigorous integration of quantitative and qualitative findings through joint display matrices.
- Provide methodological guidance for implementing convergent parallel designs in resource-limited healthcare settings.

## 2 Methods

### 2.1 Study design

We employed a convergent parallel mixed methods design with equal priority given to quantitative and qualitative components (12–14, 92). Both components addressed the same research questions from complementary perspectives, with data collection occurring simultaneously over a 12-month period (January–December 2023). Integration occurred during analysis and interpretation phases through systematic comparison using joint display matrices (14, 90–92). This design was selected because the research questions required both breadth (exploring associations across many participants) and depth (understanding mechanisms and contextual factors), and because we aimed to triangulate findings to enhance validity (80, 81).

### 2.2 Setting and participants

#### 2.2.1 Quantitative component

The study was conducted across 36 ART clinics in Ghana’s Ashanti Region, including teaching hospitals, district hospitals, health centres, and Community-based Health Planning and Services (CHPS) compounds. This facility diversity enabled examination of adherence patterns across different levels of healthcare infrastructure.

The Ashanti Region is Ghana’s most populous administrative region with over five million inhabitants and an estimated HIV prevalence of 2.3%, which is above the national average of 1.7% (21, 22). The region encompasses predominantly urban and peri-urban settlements concentrated around the regional capital, Kumasi, though rural communities with limited healthcare access are also represented. The region operates a well-established network of ART service delivery points managed under the Ghana Health Service, ranging from tertiary-level hospitals with dedicated HIV clinics to community-level CHPS compounds where ART is dispensed by community health officers. Socioeconomically, the Ashanti Region exhibits significant heterogeneity, with a large informal trading sector, agricultural communities in outlying districts, and a substantial population of employed and self-employed individuals in urban Kumasi. This heterogeneity is directly relevant to the adherence variables examined in this study, particularly employment status, transportation access, and economic resources, as it creates a wide spectrum of structural barriers and facilitators within a single geographic study area (21, 22, 27, 48).

##### 2.2.1.1 Sample

The analytical sample comprised 288 participants with complete adherence measurement data, recruited from 36 facilities during July–December 2023.

##### 2.2.1.2 Sampling strategy

We employed purposive sampling to recruit patients across the healthcare facilities (78). Specifically, recruitment deliberately targeted patients who met the following criteria: (1) documented evidence of at least one missed clinic appointment for medication refill during the preceding 12 months, indicating adherence challenges, and (2) subsequent successful re-engagement with ART services, demonstrated by attending the current clinic visit. This purposive sampling approach was intentional and strategically designed to ensure adequate variation in adherence levels for statistical exploration while concentrating investigation on patients experiencing suboptimal adherence patterns.

##### 2.2.1.3 Rationale for purposive sampling

HIV and AIDS is associated with substantial stigma, and people living with HIV often distance themselves from activities that might expose their HIV status (33–35, 93–95). This social context makes probability sampling extremely challenging. Additionally, we sought specifically to understand adherence barriers among patients who had experienced documented non-adherence episodes but maintained some engagement with care, rather than conducting a general population survey. This focused sampling enables in-depth investigation of factors associated with partial adherence among patients demonstrating both adherence challenges and continued healthcare engagement.

##### 2.2.1.4 Implications for generalizability

This purposive sampling strategy has critical implications. Findings apply specifically to patients with documented missed appointments who successfully re-engaged, not to consistently adherent patients without missed appointments, not to patients lost to follow-up who stopped attending clinic entirely, and not to the general population of people living with HIV in the Ashanti Region. Adherence rates, barrier profiles, and intervention needs likely differ substantially across these distinct subpopulations. Results should not be extrapolated to estimate population-level adherence prevalence or to characterize all HIV-positive individuals in the region.

##### 2.2.1.5 Inclusion criteria

(1) age 18 years or older, (2) receiving ART for at least 6 months, (3) documented evidence of at least one missed clinic appointment for medication refill during the preceding 12 months, (4) current re-engagement with ART services, (5) willing to provide informed consent, and (6) availability of seven-day pill count adherence data at the current clinic visit, which required patients to present their medication containers for counting.

##### 2.2.1.6 Exclusion criteria

(1) severe cognitive impairment precluding informed consent, (2) active psychiatric illness requiring hospitalization, and (3) inability to communicate in English, Akan (Twi), or Fante.

#### 2.2.2 Qualitative component

We conducted 20 in-depth semi-structured interviews: 10 with patients living with HIV who had experienced at least one missed clinic appointment for medication refill but had successfully re-engaged with ART services, and 10 with healthcare providers (4 physicians, 3 nurses, 2 pharmacists, 1 counselor) involved in ART service delivery. This missed appointment criterion was consistently applied to both patient and provider recruitment: patient participants were selected on the basis of documented missed appointments in their clinic records, while provider participants were purposively selected from clinics where such patients were being managed. Purposive sampling ensured diversity in age, gender, education level, employment status, and facility type (78). Recruitment continued until thematic saturation was achieved, indicated by emergence of no new themes in the final three interviews (96, 97). Interview duration ranged from 25 to 55 min (mean 38 min).

### 2.3 Data collection

#### 2.3.1 Quantitative data collection

Trained research assistants administered structured questionnaires in clinic rooms that ensured privacy during scheduled ART visits (98, 99). The questionnaire assessed sociodemographic characteristics (gender, age group, marital status, education level, employment status) and theoretical constructs including behavioral skills, self-efficacy beliefs, self-care strategies, social support factors, economic factors, and facility factors using validated scales (63–68).

##### 2.3.1.1 Adherence measurement

seven-day pill count adherence was conducted at scheduled monthly clinic visits (100–103). Research assistants asked patients to bring all medication containers, counted remaining pills, verified prescription records, and calculated adherence percentage as: (Pills consumed/Pills prescribed) × 100 for the 7 days immediately prior to the visit. This seven-day recall window balances recency (reducing recall bias) with adequate duration to detect non-adherence patterns (73, 88, 102, 103).

##### 2.3.1.2 Antiretroviral regimens

Ghana’s national treatment guidelines specify three-drug combinations (21):

- Efavirenz-based: Tenofovir/Lamivudine/Efavirenz (TDF/3TC/EFV), first-line
- Dolutegravir-based: Tenofovir/Lamivudine/Dolutegravir (TDF/3TC/DTG), preferred first-line
- Zidovudine-based: Zidovudine/Lamivudine/Nevirapine (AZT/3TC/NVP), second-line

Adherence was calculated separately for each regimen type. Mean adherence across the patient’s prescribed regimen served as the primary continuous outcome variable for quantitative analyses.

#### 2.3.2 Qualitative data collection

Semi-structured interview guides were developed based on theoretical frameworks and literature review, pilot-tested with three participants, and refined based on feedback (76, 77). Patient interviews explored: experiences with ART adherence, specific challenges encountered, coping strategies employed, support systems available, economic and transportation barriers, stigma and disclosure issues, healthcare facility experiences, and suggestions for improving adherence support. Provider interviews examined: observations of patient adherence patterns, perceived barriers to adherence, facility-level challenges, support systems implemented, communication practices, medication stock-out experiences, and recommendations for system-level improvements (59, 84, 85, 89).

Interviewers used probing questions to elicit detailed examples and clarify ambiguous responses. Field notes documented non-verbal cues, interview context, and initial impressions. All interviews were conducted in confidential settings at healthcare facilities or private community locations based on participant preference (76, 77, 104, 105). All interviews were audio-recorded with permission, transcribed verbatim, and translated into English with back-translation verification.

### 2.4 Data analysis

#### 2.4.1 Quantitative analysis

##### 2.4.1.1 Composite score calculation

Composite scores were calculated as means of constituent items for each theoretical construct (71–73). This approach is standard when using validated theoretical frameworks, as individual items are designed to measure latent constructs. Composite scores reduce measurement error, improve reliability through aggregation across multiple indicators, and reduce multicollinearity that would be substantially worse with 51 separate predictors in regression models. All scales were coded such that higher scores indicate worse status (more problems, fewer resources, poorer outcomes).

##### 2.4.1.2 Descriptive statistics

Frequencies and percentages summarized categorical variables. Means and standard deviations described continuous variables. Missing data patterns were examined for all variables.

##### 2.4.1.3 Bivariate analyses

Independent samples *t*-tests compared mean adherence rates between groups for binary predictors. Chi-square tests examined associations between categorical demographic variables and adherence.

##### 2.4.1.4 Multilevel regression

We employed multilevel linear regression with random intercept for facility to account for clustering of the 288 participants within 36 healthcare facilities (106, 107). The model included sociodemographic predictors and theoretical construct composite scores as fixed effects, with facility as a random effect. Intraclass correlation coefficient (ICC) quantified the proportion of total variance attributable to facility-level clustering. This approach partitions variance into between-facility and within-facility components while providing appropriate standard errors (106–109).

##### 2.4.1.5 Model diagnostics

Variance inflation factors (VIF) assessed multicollinearity, with VIF greater than 10 indicating problematic correlation (110, 111). Residual plots examined normality, linearity, and homoscedasticity assumptions (112, 113).

All quantitative analyses used SPSS Version 26.0. Statistical significance was set at p less than 0.05 (two-tailed).

#### 2.4.2 Qualitative analysis

Thematic analysis followed Braun and Clarke’s six-phase approach (114, 115): (1) familiarization through repeated reading of transcripts, (2) systematic generation of initial codes, (3) searching for themes by collating codes, (4) reviewing themes for coherence and distinctiveness, (5) defining and naming themes, and (6) selecting illustrative quotations.

##### 2.4.2.1 Coding procedures

Two independent coders (VLD and EO) coded 25% of transcripts (5 of 20 interviews). Initial agreement was 82% at the code level. Disagreements were resolved through discussion until consensus was reached, and the refined coding structure was applied to remaining transcripts by the primary coder with regular team meetings for ongoing reliability checks.

##### 2.4.2.2 Intercoder reliability

Cohen’s Kappa for major thematic categories: Economic barriers kappa = 0.79 (substantial agreement), Stigma/disclosure kappa = 0.85 (almost perfect), Facility factors kappa = 0.73 (substantial), Individual factors kappa = 0.81 (almost perfect), Social support kappa = 0.77 (substantial). These metrics exceed conventional thresholds (kappa greater than 0.70), indicating trustworthy coding (116, 117).

##### 2.4.2.3 Software

NVivo 12 (QSR International, Melbourne, Australia) facilitated data organization, coding, and theme development.

##### 2.4.2.4 Trustworthiness strategies

Credibility was enhanced through investigator triangulation (multiple coders), data source triangulation (patients and providers), and member checking with a subset of participants (104, 105). Transferability was addressed through thick description of context. Dependability was ensured through detailed audit trail documenting analytical decisions. Confirmability was supported by reflexive journaling acknowledging researcher perspectives and potential biases (104, 105).

#### 2.4.3 Integration

Integration occurred through joint display matrices systematically comparing quantitative results with qualitative themes (90, 91). We created separate matrices for major adherence domains, with columns presenting quantitative associations, qualitative themes with representative quotes, and integration interpretation noting convergence, expansion, or divergence. The integration process followed systematic steps outlined in published mixed methods guidance (10, 11, 14, 79, 90–92).

### 2.5 Ethical considerations

This study received ethical approval from the University of Cape Coast Institutional Review Board (approval number: ET/HTP/18/0002) and the Ghana Health Service Ethics Review Committee (approval number: GHS-ERC:027/08/22). Written informed consent was obtained from all participants. The study was conducted in accordance with the Declaration of Helsinki and Ghana’s national guidelines for research involving human participants. Participants were informed of their right to withdraw at any time without consequence to medical care or employment status. Confidentiality was maintained through use of unique identification codes, secure data storage, and restricted data access.

## 3 Results

### 3.1 Sociodemographic characteristics

Table 1 presents the sociodemographic characteristics of the 288 participants included in the analytical sample. The sample was predominantly female (66.0%, *n* = 190), consistent with Ghana’s national HIV care data indicating that women constitute approximately 65% of individuals enrolled in ART programs nationally (19, 21). Age distribution showed 41.0% (*n* = 118) were aged 18–35 years, 38.2% (*n* = 110) were 36–50 years, and 20.8% (*n* = 60) were over 50 years. Marital status varied, with 45.8% (*n* = 132) married, 28.5% (*n* = 82) single, 16.0% (*n* = 46) divorced/separated, and 9.7% (*n* = 28) widowed. Educational attainment showed 41.0% (*n* = 118) had completed primary education only, 34.0% (*n* = 98) had Junior High School/Middle School Leaving Certificate (JHS/MSLC), 20.8% (*n* = 60) had Secondary/Technical education, and 4.2% (*n* = 12) had no formal education. Employment status indicated that 58.3% (*n* = 168) were employed, while 41.7% (*n* = 120) were unemployed.

**Table 1:** Sociodemographic characteristics (*N* = 288).

Characteristic	Category	Frequency	Percentage
Gender	Male	98	34.0%
	Female	190	66.0%
Age group	18–35 years	118	41.0%
	36–50 years	110	38.2%
	>50 years	60	20.8%
Marital status	Single	82	28.5%
	Married	132	45.8%
	Divorced/separated	46	16.0%
	Widowed	28	9.7%
Education level	No formal education	12	4.2%
	Primary	118	41.0%
	JHS/MSLC	98	34.0%
	Secondary/technical	60	20.8%
Employment status	Employed	168	58.3%
	Unemployed	120	41.7%

The educational profile reflects broader literacy patterns in the Ashanti Region, where universal primary education has expanded access to schooling but completion of secondary-level programs remains limited for a substantial proportion of the adult population (20, 21). The relatively high employment rate (58.3%) among participants likely reflects the specific subpopulation recruited, namely patients who maintained sufficient healthcare engagement to attend clinic visits and present pill count data, who may be more economically stable than those who disengage from care entirely.

### 3.2 Adherence rates by antiretroviral regimen

Among the 288 participants with seven-day pill count data, mean adherence rates were suboptimal across all three antiretroviral regimen types. For efavirenz-based regimens (*n* = 105), mean adherence was 80.29% (SD = 20.55%, range 14.29–100%). Dolutegravir-based regimens (*n* = 98) showed mean adherence of 79.00% (SD = 22.73%, range 0–100%). Zidovudine-based regimens (*n* = 85) demonstrated mean adherence of 78.58% (SD = 23.06%, range 0–100%). All three mean adherence rates fell substantially below the 95% threshold historically considered necessary for optimal viral suppression, though modern integrase inhibitor-based regimens may tolerate somewhat lower adherence while still achieving viral load below 1,000 copies/mL per the WHO 2023 definition (7–9, 15) (see Table 2).

**Table 2:** Seven-day pill count adherence rates by regimen (*N* = 288).

Regimen type	*n*	Mean (%)	SD (%)	Median (%)	Range (%)	<80% *n* (%)	80–94% *n* (%)	> = 95% *n* (%)
Efavirenz-based (TDF/3TC/EFV)	105	80.29	20.55	85.71	14.29–100	41 (39.0%)	35 (33.3%)	29 (27.6%)
Dolutegravir-based (TDF/3TC/DTG)	98	79.00	22.73	85.71	0–100	40 (40.8%)	32 (32.7%)	26 (26.5%)
Zidovudine-based (AZT/3TC/NVP)	85	78.58	23.06	85.71	0–100	36 (42.4%)	26 (30.6%)	23 (27.1%)
Overall	288	79.29	21.45	85.71	0–100	117 (40.6%)	93 (32.3%)	78 (27.1%)

Adherence calculated as (pills consumed/pills prescribed) × 100 over 7 days immediately preceding clinic visit. TDF, Tenofovir; 3TC, Lamivudine; EFV, Efavirenz; DTG, Dolutegravir; AZT, Zidovudine; NVP, Nevirapine. Categories: less than 80% indicates poor adherence; 80–94% indicates suboptimal adherence; 95% or above indicates optimal adherence.

The distribution of adherence levels reveals concerning patterns: only 27.1% (*n* = 78) of participants achieved optimal adherence (95% or above) across all regimens. Approximately one-third (32.3%, *n* = 93) demonstrated suboptimal adherence in the 80–94% range, while 40.6% (*n* = 117) exhibited poor adherence below 80%, a level associated with a high probability of virologic failure according to WHO guidance (3, 4, 9, 15). These adherence figures are consistent with the purposive sampling design, which specifically recruited patients with documented missed clinic appointments, resulting in an expected shift of the adherence distribution toward lower values relative to a general ART clinic population.

### 3.3 Composite scores for theoretical constructs

Table 3 presents descriptive statistics for the six composite scores derived from theoretical frameworks (63–68, 71, 72). All composites were coded such that higher scores indicate worse status.

**Table 3:** Descriptive statistics for composite scores (*N* = 288).

Composite variable	Items	Scale range	Mean	SD	Median	Cronbach’s alpha
Behavioral skills	5	1.00–2.00	1.02	0.02	1.00	0.68
Self-efficacy	8	1.00–5.00	2.12	0.68	2.00	0.84
Self-care strategies	12	1.00–2.00	1.18	0.09	1.17	0.72
Social support	5	1.00–5.00	2.28	0.74	2.20	0.81
Economic factors	5	1.00–5.00	2.64	0.72	2.60	0.79
Facility factors	5	1.00–5.00	2.42	0.68	2.40	0.83

All composite scores coded such that higher values indicate worse status (more problems, fewer resources, lower adherence support). Scale Range indicates possible minimum and maximum scores. Internal consistency reliability (Cronbach’s alpha) was acceptable for all scales (alpha 0.68 or above) (6).

Internal consistency reliability was acceptable for most scales (Cronbach’s alpha = 0.72–0.84), with only Behavioral Skills showing borderline adequacy (alpha = 0.68) (6). The Behavioral Skills composite demonstrated extremely low standard deviation (SD = 0.02), indicating restricted range that limits discriminatory power. The mean Economic Factors composite score of 2.64 (SD = 0.72), the highest among all constructs, suggests that economic challenges are perceived as more burdensome than other factors within this sample. Social Support (Mean = 2.28, SD = 0.74) and Facility Factors (Mean = 2.42, SD = 0.68) also registered above the midpoint of their respective scales, pointing to moderate perceived deficits in these domains.

### 3.4 Bivariate associations

Table 4 presents bivariate associations between sociodemographic characteristics and mean adherence rates. Significant associations were observed for gender, employment, education, and marital status; however, these unadjusted results should be interpreted with caution given the confounding relationships addressed in the multivariate model. Gender demonstrated a significant bivariate difference (*t* = 3.68, *p* < 0.001), with males reporting higher mean adherence (84.52%, SD = 18.22%) compared to females (76.47%, SD = 22.51%). Employment status showed the strongest bivariate association (*t* = 5.24, *p* < 0.001), with employed participants demonstrating substantially higher adherence (84.73%, SD = 18.65%) than unemployed participants (71.58%, SD = 23.12%). Education level showed significant variation (*F* = 5.12, *p* = 0.002), with JHS/MSLC-educated participants showing highest adherence (85.92%, SD = 17.88%). Marital status showed modest variation (*F* = 2.94, *p* = 0.034). These bivariate patterns differ substantially from multivariate findings, underscoring the importance of confounder adjustment and the limitations of bivariate analysis in complex multi-predictor contexts.

**Table 4:** Bivariate associations between demographics and adherence (*N* = 288).

Variable	Category	*n*	Mean Adherence (%)	SD	Test Statistic	*p*-value
Gender	Male	98	84.52	18.22	*t* = 3.68	<0.001
	Female	190	76.47	22.51		
Employment	Employed	168	84.73	18.65	*t* = 5.24	<0.001
	Unemployed	120	71.58	23.12		
Education	No formal education	12	73.81	24.68	*F* = 5.12	0.002
	Primary	118	75.64	22.45		
	JHS/MSLC	98	85.92	17.88		
	Secondary/technical	60	78.15	21.33		
Marital Status	Single	82	82.89	19.45	*F* = 2.94	0.034
	Married	132	76.12	22.18		
	Divorced/separated	46	76.85	21.95		
	Widowed	28	84.29	18.74		

Table 5 presents correlations between composite scores and adherence. Self-Care Strategies demonstrated the strongest correlation (*r* = −0.68, *p* < 0.001), Self-Efficacy showed strong negative correlation (*r* = −0.51, *p* < 0.001), Social Support correlated significantly (*r* = −0.48, *p* < 0.001), and Facility Factors showed moderate negative correlation (*r* = −0.44, *p* < 0.001). Economic Factors showed a counterintuitive positive correlation (*r* = 0.18, *p* = 0.002).

**Table 5:** Correlations between composite scores and adherence (*N* = 288).

Composite variable	Pearson r	*p*-value
Behavioral skills	−0.42	<0.001
Self-efficacy	−0.51	<0.001
Self-care strategies	−0.68	<0.001
Social support	−0.48	<0.001
Economic factors	0.18	0.002
Facility factors	−0.44	<0.001

All correlations significant at *p* < 0.05. Composite scores coded such that higher values indicate worse status. Negative correlations indicate theoretically expected patterns (24, 73, 118). The positive correlation for Economic Factors is counterintuitive and is examined in the context of multicollinearity in section 3.5.

### 3.5 Multilevel regression analysis

Table 6 presents the multilevel linear regression model examining associations between demographic factors, theoretical constructs, and mean adherence rates, accounting for facility clustering (106, 107). This model provides confounder-adjusted estimates and should be given greater interpretive weight than the bivariate results presented in section 3.4. Variance inflation factors (VIF) revealed severe multicollinearity for theoretical constructs: Self-Care Strategies (VIF = 379.19), Economic Factors (VIF = 39.65), Facility Factors (VIF = 22.73), Social Support (VIF = 18.64), and Self-Efficacy (VIF = 12.48). These values substantially exceed the conventional threshold of VIF = 10 for problematic multicollinearity (110, 111, 113). Demographic variables showed acceptable multicollinearity (VIF range: 1.82–4.65).

**Table 6:** Multilevel linear regression model predicting adherence (*N* = 280, 36 facilities).

Predictor	*B*	SE	95% CI	*p*-value	VIF
Demographics					
Male gender	−8.64	2.12	[−12.80, −4.48]	<0.001	1.82
Married (vs single)	7.18	2.56	[2.16, 12.20]	0.005	3.24
Divorced (vs single)	8.35	2.94	[2.59, 14.11]	0.005	2.89
JHS/MSLC (vs none)	−20.18	4.28	[−28.58, −11.78]	<0.001	3.18
Secondary/tech (vs none)	5.94	2.75	[0.54, 11.34]	0.031	2.94
Employed	−13.21	2.38	[−17.88, −8.54]	<0.001	4.65
Theoretical constructs					
Behavioral skills	−27.32	8.12	[−43.25, −11.39]	0.001	8.92
Self-efficacy	−10.89	2.64	[−16.06, −5.72]	<0.001	12.48
Self-care strategies	−96.48	12.84	[−121.68, −71.28]	<0.001	379.19
Social support	−22.53	3.18	[−28.77, −16.29]	<0.001	18.64
Economic factors	4.79	1.42	[2.01, 7.57]	0.001	39.65
Facility factors	−15.82	2.92	[−21.54, −10.10]	<0.001	22.73
Model fit					
ICC	0.040				
Marginal *R*-squared	0.695				
Conditional *R*-squared	0.711				

B, unstandardized coefficient; SE, standard error; CI, confidence interval; VIF, variance inflation factor. *N* = 280 due to 8 participants with missing covariate data. ICC, intraclass correlation (proportion of variance between facilities). Severe multicollinearity (VIF>10 for most constructs) limits coefficient interpretation (110–113).

The intraclass correlation coefficient (ICC) = 0.040 indicates that only 4.0% of total adherence variance occurs between facilities, while 96.0% occurs within facilities (106, 107). The marginal R-squared = 0.695 indicates that fixed effects explain 69.5% of adherence variance, while the conditional R-squared = 0.711 reflects the combined contribution of fixed and random effects.

Among the demographic predictors with acceptable multicollinearity, male gender was associated with 8.64 percentage points lower adherence after adjustment (*B* = −8.64, *p* < 0.001), a finding that reverses the direction observed in bivariate analysis and is examined in detail in the discussion. Employment was also associated with lower adherence after adjustment (*B* = −13.21, *p* < 0.001), reversing the strong positive bivariate relationship. Married and divorced marital statuses were associated with higher adherence relative to single status (*B* = 7.18, *p* = 0.005 and *B* = 8.35, *p* = 0.005, respectively). Among educational levels, JHS/MSLC education was associated with lower adherence relative to no formal education (*B* = −20.18, *p* < 0.001). For theoretical constructs, the severe multicollinearity affecting Self-Care Strategies (VIF = 379.19), Economic Factors (VIF = 39.65), Facility Factors (VIF = 22.73), and Social Support (VIF = 18.64) renders their individual regression coefficients unreliable; these should not be interpreted in isolation and are addressed through mixed methods integration in section 3.7. Self-Efficacy (VIF = 12.48) and Behavioral Skills (VIF = 8.92) are somewhat more interpretable, with both showing negative associations with adherence (*B* = −10.89, *p* < 0.001 and *B* = −27.32, *p* = 0.001, respectively), consistent with theoretical predictions that lower self-efficacy and weaker behavioral skills are associated with poorer adherence outcomes (63–66).

### 3.6 Qualitative findings

Thematic analysis of 20 in-depth interviews identified five major themes (114, 115).

#### 3.6.1 Theme 1: economic barriers to adherence

Participants described profound economic constraints affecting medication adherence through multiple pathways (25–28, 43). Transportation costs emerged as a dominant barrier, with patients reporting fares of 20–40 Ghana Cedis (approximately $3–6 USD) for round-trip clinic visits representing substantial household expenditure (25, 27). One patient explained:

“Sometimes I don’t have the money for transport. The clinic is far and I need about 30 cedis. If I don’t have it, I miss my appointment and my drugs finish.” (PT-03)

Food insecurity compounded adherence challenges, as antiretroviral medications often cause nausea when taken on an empty stomach (28, 43–46). A patient described:

“The drugs make me very sick if I don’t eat. But sometimes there is no food at home. So I skip the drugs because I know I will vomit.” (PT-07)

Healthcare providers corroborated these economic barriers:

“Many of our patients are very poor. They tell us they cannot afford transport to come for refills. Some sell their medications to buy food.” (HCP-02)

#### 3.6.2 Theme 2: HIV-related stigma and disclosure concerns

Stigma emerged as a pervasive barrier operating at internalized, interpersonal, and structural levels (33–37). Patients described concealing medications to avoid inadvertent HIV status disclosure:

“I hide my drugs in a bag. If my family sees them, they will know I have the disease. So sometimes I forget to take them when I am outside.” (PT-05)

Fear of workplace stigma created specific adherence barriers for employed participants. One patient described the dilemma of taking medications during work hours:

“At work, I cannot take my drugs openly. I have to sneak to the toilet. Sometimes meetings are long and I miss the time.” (PT-09)

Healthcare providers identified structural stigma within facilities as an underappreciated barrier:

“Some of my colleagues still make patients feel embarrassed. When patients feel judged, they don’t come back.” (HCP-06)

#### 3.6.3 Theme 3: healthcare facility factors

Medication stock-outs emerged as a recurring structural challenge, with providers describing national supply chain disruptions creating gaps in medication availability:

“Sometimes we run out of TDF/3TC/DTG. Patients come all the way, wait for hours, and go home without drugs. After that some don’t come back.” (HCP-04)

Patients described long wait times as a significant deterrent to consistent clinic attendance, particularly for employed participants:

“I wait from morning until afternoon sometimes. My employer won’t let me keep missing work. So I try to skip appointments when I can.” (PT-06)

Quality of provider-patient interaction emerged as a facilitating factor when positive. Patients who described supportive and respectful provider relationships reported higher motivation to maintain clinic attendance.

#### 3.6.4 Theme 4: individual motivation and self-efficacy

Perceived personal vulnerability and understanding of treatment benefits emerged as protective motivators (67–70). Patients who had experienced illness prior to ART initiation described strong personal motivation:

“I was very sick before I started the drugs. I know what it feels like without them. So I try hard to take them every day, even when it is difficult.” (PT-02)

Conversely, patients who felt well and perceived themselves as no longer at risk described motivation challenges:

“Sometimes I feel so healthy I think I don’t need the drugs anymore. Then I stop for a few days.” (PT-04)

#### 3.6.5 Theme 5: social support

Disclosure to trusted family members and the presence of treatment supporters facilitated adherence through practical assistance and emotional encouragement (50–53):

“My wife helps me remember. She prepares food before I take the drugs. I never miss when I am at home.” (PT-01)

Participants without disclosed status described isolation that compounded adherence difficulties:

“Nobody knows except my doctor. I take the drugs alone, hiding. When I travel, there is nobody to remind me.” (PT-08)

### 3.7 Integration through joint display matrices

Tables 7–9 present joint display matrices that integrate findings on economic barriers, healthcare facility factors, and HIV-related stigma and social support.

**Table 7:** Joint display matrix for economic barriers.

Quantitative finding	Qualitative theme	Integration interpretation
Economic factors composite: mean = 2.64, highest among constructs	Transportation costs, food insecurity, lost wages as dominant barriers	Convergence: both components identify economic burden as primary adherence impediment
Positive regression coefficient (*B* = 4.79, VIF = 39.65)	All interviewed patients described economic barriers; providers confirmed as root cause of missed appointments	Divergence resolved: coefficient unreliable due to severe multicollinearity; qualitative evidence overrides anomalous quantitative finding
Employment associated with lower adherence in multivariate model (*B* = −13.21)	Employed patients described time conflicts, workplace stigma, and inflexible clinic hours as barriers	Expansion: qualitative data explains mechanisms by which employment creates adherence challenges after controlling for economic resources

**Table 8:** Joint display matrix for healthcare facility factors.

Quantitative finding	Qualitative theme	Integration interpretation
Facility Factors: mean = 2.42; significant regression coefficient *B* = −15.82, *p* < 0.001	Stock-outs, long wait times, provider attitudes as barriers; supportive providers as facilitators	Convergence: patients perceiving worse facility factors show lower adherence; qualitative data identifies specific mechanisms
ICC = 0.040: 96% of variance within facilities	Episodic stock-outs and variable wait times described within the same facility on different visits	Expansion: low ICC explained by episodic, time-varying facility challenges rather than stable structural differences between facilities

**Table 9:** Joint display matrix for HIV-related stigma and social support.

Quantitative finding	Qualitative theme	Integration interpretation
Social Support composite: *r* = −0.48, *B* = −22.53 (VIF = 18.64)	Stigma creates concealment behaviors that disrupt adherence routines; social support from family members facilitates medication-taking	Convergence: theoretically expected association supported by qualitative mechanisms; VIF limits coefficient precision but direction confirmed
Gender: male associated with lower adherence in multivariate model (*B* = −8.64)	Male participants described workplace stigma, disclosure concerns, and masculinity norms as barriers	Expansion: qualitative data provides mechanistic explanation for gender disparity observed in multivariate model

## 4 Discussion

### 4.1 Principal findings

This convergent parallel mixed methods study provides evidence on ART adherence barriers among 288 patients with documented missed appointments who re-engaged in care across 36 facilities in Ghana’s Ashanti Region. Mean adherence rates of 78–80% fell below the 95% threshold historically associated with optimal viral suppression (7, 8, 15), and below the level at which published studies from Ghana and comparable West African settings have consistently demonstrated virologic suppression rates of 60–70% for efavirenz-based regimens and 70–80% for dolutegravir-based regimens (9, 16, 17). Given the heterogeneous socioeconomic characteristics of the Ashanti Region described in the methods section, including significant inequality between urban Kumasi and peri-urban and rural districts, adherence barriers are likely to manifest differently across patients within the same study area, a contextual factor that the ICC analysis and qualitative data both illuminate.

The primary substantive contribution of the study is convergent evidence that multilevel barriers, including economic constraints, HIV-related stigma, healthcare facility challenges, variable social support, and individual motivational factors, interact to shape adherence among this specific subpopulation (23, 24, 86, 87, 118). The primary methodological contribution is the rigorous implementation and transparent reporting of a convergent parallel design, including systematic integration through joint display matrices and an explicit framework for resolving quantitative-qualitative divergence.

Interpretation of this study should prioritize findings from the multivariate model over bivariate comparisons, as the multivariate approach controls for confounders and provides more credible estimates of independent associations. Where multicollinearity limits interpretation of specific multivariate coefficients, the joint display integration framework guides resolution using qualitative evidence and prior published work.

### 4.2 Economic barriers and the counterintuitive coefficient

The positive regression coefficient for economic factors (*B* = 4.79, *p* = 0.001) contradicts theoretical expectations and extensive empirical evidence documenting economic barriers as fundamental adherence impediments in resource-limited settings (25–28, 43, 44, 48, 49). Careful examination reveals that this anomaly results from severe multicollinearity (VIF = 39.65), with economic factors sharing substantial variance with social support (*r* = 0.52), self-care strategies (*r* = 0.45), and facility factors (*r* = 0.48). When predictors share such a high proportion of variance, regression coefficients become statistically unstable and can reverse direction as a mathematical artifact (110, 111, 119, 120).

Multiple additional explanations are plausible. Patients facing the most extreme economic hardship may be systematically absent from the sample, having been unable to reach healthcare facilities at all during the recruitment period, creating restriction of range in the measured economic barrier variable. Furthermore, some patients who recognized significant economic barriers may have responded by increasing their adherence effort as a compensatory strategy, a pattern sometimes observed in health behavior research (98, 99). However, qualitative evidence is unequivocal and internally consistent across 20 independent interviews: economic constraints, including transportation costs of 20–40 Ghana Cedis per round-trip visit, food insecurity that precluded taking medications safely, and opportunity costs from missed workdays, were identified by all patient participants as primary adherence impediments, confirmed by providers.

Following established mixed methods integration principles for resolving quantitative-qualitative divergence (79, 82, 83, 91), we conclude that the qualitative evidence and the weight of prior literature from Ghana and comparable West African settings are more credible than the anomalous multicollinearity-affected coefficient. We therefore interpret economic barriers as critical impediments to adherence in this population (25, 27, 28, 43–45, 48, 49, 121–123). This interpretive decision exemplifies the methodological value of convergent parallel designs: qualitative evidence can identify and explain quantitative anomalies, preventing erroneous conclusions from statistically significant but artifactual coefficients.

### 4.3 Gender, employment, and multivariate adherence associations

Among the demographic predictors with acceptable multicollinearity in the multilevel model, male gender was significantly associated with lower adherence after adjustment (*B* = −8.64, *p* < 0.001), with employed status similarly predicting lower adherence when economic resources were controlled (*B* = −13.21, *p* < 0.001). These findings differ markedly from the bivariate patterns in the opposite directions, and warrant careful interpretation.

The gender reversal from bivariate (males higher adherence) to multivariate (males lower adherence) reflects the operation of confounders. In bivariate analysis, male gender correlates with employment and economic resources, both of which correlate with adherence. Once economic and theoretical construct variables are included in the multivariate model, these confounding pathways are controlled, revealing an underlying gender disadvantage consistent with published evidence on male disengagement from HIV care in sub-Saharan Africa. Studies from Kumasi and other Ghanaian sites, as well as a multicenter cohort in South Africa by Cornell and colleagues (16), document that men show lower retention in care and worse virologic outcomes than women at comparable adherence levels, partly due to more advanced disease at ART initiation, masculinity norms that discourage healthcare engagement, and workplace disclosure concerns (16, 124–128). Our qualitative findings specifically corroborate the workplace stigma and time-conflict mechanisms, with male participants describing fears that taking medications visibly at work would expose their HIV status to employers and colleagues.

The employment paradox in the multivariate model, specifically that employed status predicts lower adherence (*B* = −13.21) after controlling for economic factors, reflects a suppression effect that is fully explained by qualitative data. In bivariate analysis, employment functions as a proxy for economic stability, producing an apparent 13-percentage-point adherence advantage. Once economic resources are directly measured and included in the model, the confounding economic pathway is controlled, exposing the countervailing adherence costs of employment: conflicts between ART clinic hours and workplace schedules, stigma concerns about medication-taking visibility in occupational settings, and inflexible work arrangements. Published studies from sub-Saharan Africa document precisely this duality, with some reporting protective economic effects of employment and others identifying time-constraint barriers, differences that likely reflect whether or not economic resources were controlled in the analysis (25, 44, 48, 49, 127–129).

### 4.4 Facility factors and the modest intraclass correlation coefficient (ICC)

The ICC = 0.040, indicating that only 4.0% of total adherence variance occurs between facilities, initially appears to contradict the significant facility factors regression coefficient (*B* = −15.82, *p* < 0.001) and the prominent qualitative themes about stock-outs, wait times, and provider quality (17, 29–32). However, careful consideration reveals these findings are compatible and together illustrate a key insight made possible by mixed methods integration (90, 91, 106, 107).

The facility factors composite assessed patient perceptions of medication education quality, provider treatment, information provision, competence, and problem management. These items capture healthcare quality experiences that vary within facilities over time and across different providers at the same facility, rather than stable structural differences between facilities (130, 131). A patient may experience excellent care during one visit (well-stocked pharmacy, short wait, supportive nurse) and poor care during the next visit at the same facility when a stock-out occurs, different staff are on duty, or the patient volume is high. Qualitative data from providers directly confirmed this interpretation: episodic, nationally driven supply chain disruptions affected all facilities unpredictably; patient wait times depended on staffing levels and daily volume; and provider quality varied within facilities as different clinicians rotated through ART clinics (29, 132–135).

The significant regression coefficient (*B* = −15.82) confirms that patients who perceive worse facility quality have lower adherence, while the modest ICC confirms that these quality perceptions vary primarily within facilities rather than between them. Both findings are valid and complementary, and their apparent contradiction is resolved by qualitative evidence on the episodic, time-varying nature of facility challenges. This pattern, where within-facility temporal variability rather than between-facility structural differences drives adherence outcomes, has important practical implications: pharmacy supply chain stability and appointment management systems are potentially modifiable targets that could improve adherence uniformly across all facility types.

### 4.5 Stigma, social support, and self-efficacy

HIV-related stigma emerged as a cross-cutting theme shaping adherence through multiple interacting pathways. In the multivariate model, social support showed a theoretically expected negative association (*B* = −22.53, *p* < 0.001), though the high VIF (18.64) limits coefficient precision. Qualitative evidence provides clear mechanistic explanation that is consistent with published literature from Ghana and sub-Saharan Africa more broadly: internalized stigma leads to medication concealment that disrupts adherence routines; interpersonal stigma from family and community members constrains disclosure necessary for obtaining treatment support; and workplace stigma creates time and visibility barriers particularly relevant to employed male participants (33–38, 136, 137).

Self-efficacy showed a robust negative association with lower adherence in the multivariate model (*B* = −10.89, *p* < 0.001, VIF = 12.48), consistent with IMB and HBM framework predictions (63, 64, 67, 68). Qualitative data reinforced this finding: patients who described strong belief in the importance of their medications and in their own ability to manage adherence challenges reported concrete coping strategies, including reminders, treatment supporters, and proactive communication with providers. In contrast, patients with lower self-efficacy described fatalistic attitudes and passive responses to adherence barriers. This convergence between the multivariate model and qualitative findings strengthens the inference that self-efficacy is a legitimate independent predictor of adherence in this population, even acknowledging the elevated VIF.

Published cohort data from Kumasi and Ghanaian national health surveys indicate that patients with lower perceived self-efficacy and weaker social networks have consistently higher rates of missed appointments and virologic non-suppression, suggesting these constructs operate as both proximal and distal determinants of treatment outcomes in the Ghanaian ART cascade (16, 21, 52–55).

### 4.6 Limitations

Several limitations constrain interpretation and generalizability of findings.

#### 4.6.1 Sample and generalizability

The purposive sampling strategy recruiting patients with documented missed appointments who re-engaged in care creates a specific population distinct from consistently adherent patients and from those lost to follow-up entirely (41, 59–62, 78). Findings apply to patients experiencing partial adherence challenges while maintaining some healthcare engagement. Adherence rates, barrier profiles, and intervention needs likely differ substantially across these distinct subpopulations. The analytical sample of 288 participants from 36 facilities provides limited statistical power for detecting small effects and examining complex interactions.

#### 4.6.2 Absence of clinical baseline and outcome data

CD4 counts, WHO clinical staging at ART initiation, and viral load suppression status were not collected. This precludes characterization of participants by baseline immune status, assessment of immune reconstitution trajectories, or determination of whether observed adherence patterns achieved viral load suppression below 1,000 copies/mL per the WHO 2023 definition (9, 16, 17, 124, 125). Published literature consistently demonstrates that patients initiating ART with advanced HIV disease require higher adherence thresholds for virologic success and face distinct barrier profiles compared to those starting treatment earlier (16, 17, 124, 125), creating unmeasured heterogeneity in this sample. Future research must integrate behavioral adherence measurement with routine clinical monitoring.

#### 4.6.3 Measurement limitations

Seven-day pill count adherence, while more objective than self-report, remains susceptible to manipulation if patients anticipate the count (100–103). The restricted range in the Behavioral Skills composite (SD = 0.02) suggests ceiling effects. Cross-sectional design precludes causal inference. Theoretical construct measures, while showing acceptable internal consistency, demonstrated severe multicollinearity fundamentally limiting interpretation of independent effects (110–113, 119, 120, 138, 139).

#### 4.6.4 Qualitative limitations

Twenty interviews provide rich data for thematic exploration but cannot establish prevalence of identified barriers (76, 77, 96, 97). Social desirability bias may affect both survey and interview responses (98, 99). Member checking and investigator triangulation enhanced credibility, and reflexivity about researcher positionality was maintained throughout (104, 105).

### 4.7 Implications for research and practice

#### 4.7.1 Research recommendations

Future studies should employ representative probability sampling enabling population-level inference about adherence determinants. Integration of behavioral adherence measurement with routine viral load monitoring would clarify adherence-suppression relationships and identify adherence thresholds sufficient for virologic success across different regimens (7–9, 15, 118). Baseline CD4 counts or WHO staging would enable stratification by disease severity (16, 17, 124, 125). Longitudinal designs following patients over time would elucidate temporal dynamics and causal sequences (108, 109, 140–142). Ecological momentary assessment using mobile health platforms could reduce recall bias and capture real-time adherence patterns (141, 142).

#### 4.7.2 Intervention implications

The convergent evidence points to the need for multilevel interventions (60–62). Economic interventions should address transportation costs through transport vouchers or differentiated ART service delivery models, food insecurity through nutritional support or food packages with ART refills, and time constraints through flexible clinic hours and multi-month dispensing (121–123, 143, 144). Healthcare system interventions should strengthen pharmaceutical supply chains, implement patient flow management to reduce wait times, and provide facility-level training in patient-centered communication (29, 132–135). Stigma reduction approaches should include healthcare worker training on non-discriminatory practice, community education, workplace anti-discrimination policies, and peer support group facilitation (58, 136, 137, 145–149).

### 4.8 Methodological contributions

This study demonstrates key principles of rigorous convergent parallel design implementation (12–14, 80, 81, 92). Systematic integration through joint display matrices provides transparent documentation of how findings from different components relate (90, 91). The explicit framework for resolving quantitative-qualitative divergence, specifically the decision to override the anomalous economic factors regression coefficient based on credible qualitative evidence and prior published literature, addresses a critical challenge in mixed methods practice (79, 82, 83, 91). Transparent acknowledgment of severe quantitative limitations (multicollinearity, measurement range restriction, limited sample size) while still deriving substantive value from integration demonstrates that mixed methods research can generate credible insights even when individual components have limitations, provided integration is rigorous and limitations are honestly reported (110–113).

## 5 Conclusion

This study examined ART adherence among 288 patients with documented missed appointments who re-engaged in care across 36 healthcare facilities in Ghana’s Ashanti Region. Mean adherence rates of 78–80% fell below optimal levels, and multilevel barriers including economic constraints, HIV-related stigma, healthcare facility challenges, and variable social support were identified through convergent quantitative and qualitative evidence. The study’s primary contribution is methodological: demonstrating rigorous convergent parallel design implementation, including systematic joint display integration and an explicit framework for resolving quantitative-qualitative divergence. Findings are specific to patients with partial adherence challenges who maintained healthcare engagement and cannot be generalized to other HIV-positive populations. Future research should integrate behavioral adherence measurement with clinical monitoring and test multilevel interventions addressing structural and psychosocial barriers to support achievement of Ghana’s HIV treatment targets.

## Data Availability

The original contributions presented in the study are included in the article/supplementary material, further inquiries can be directed to the corresponding author.
